# The Value of Different Experimental Observables: A Transient Absorption Study of the Ultraviolet Excitation Dynamics Operating in Nitrobenzene

**DOI:** 10.1021/acs.jpca.3c02654

**Published:** 2023-07-26

**Authors:** Stuart W. Crane, Malcolm Garrow, Paul D. Lane, Kate Robertson, Alex Waugh, Jack M. Woolley, Vasilios G. Stavros, Martin J. Paterson, Stuart J. Greaves, Dave Townsend

**Affiliations:** † Institute of Photonics & Quantum Sciences, Heriot-Watt University, Edinburgh EH14 4AS, U.K.; ‡ Institute of Chemical Sciences, Heriot-Watt University, Edinburgh EH14 4AS, U.K.; § Department of Physics, University of Warwick, Coventry CV4 7AL, U.K.; ∥ School of Chemistry, University of Birmingham, Edgbaston, Birmingham B15 2TT, U.K.

## Abstract

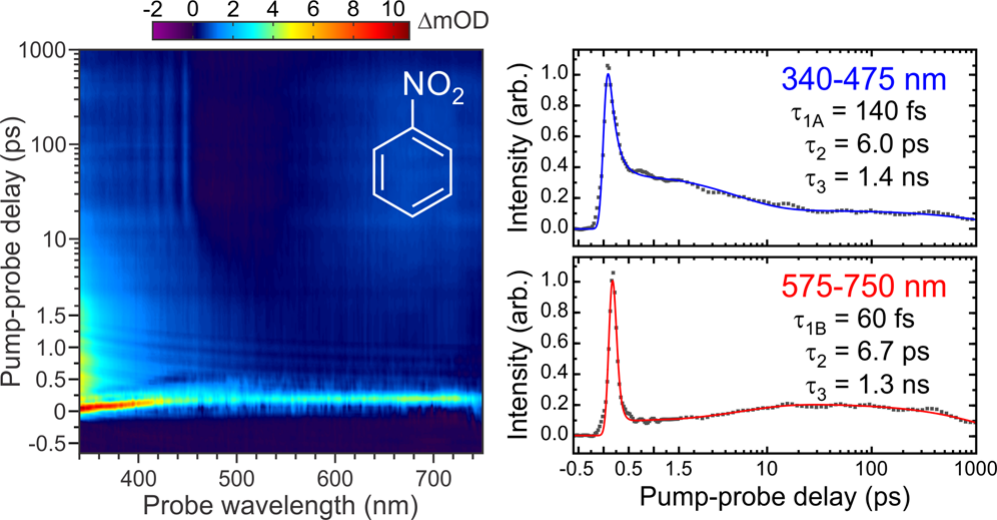

Excess energy redistribution dynamics operating in nitrobenzene under hexane and isopropanol solvation were investigated using ultrafast transient absorption spectroscopy (TAS) with a 267 nm pump and a 340–750 nm white light continuum probe. The use of a nonpolar hexane solvent provides a proxy to the gas-phase environment, and the findings are directly compared with a recent time-resolved photoelectron imaging (TRPEI) study on nitrobenzene using the same excitation wavelength [L. Saalbach et al., J. Phys. Chem. A **2021**, 125, 7174–7184]. Of note is the observation of a 1/*e* lifetime of 3.5–6.7 ps in the TAS data that was absent in the TRPEI measurements. This is interpreted as a dynamical signature of the T_2_ state in nitrobenzene—analogous to observations in the related nitronaphthalene system, and additionally supported by previous quantum chemistry calculations. The discrepancy between the TAS and TRPEI measurements is discussed, with the overall findings providing an example of how different spectroscopic techniques can exhibit varying sensitivity to specific steps along the overall reaction coordinate connecting reactants to photoproducts.

## 1 Introduction

Nitroaromatic compounds are one of the largest and most important classes of chemicals in current commercial use, with applications including pesticides, pharmaceuticals, and high-energy explosives.^1−3^ Although numerous compounds of biological origin have been identified,^4,5^ a majority are produced synthetically on an industrial scale, leading to the potential for significant environmental impact. Nitroaromatic species may also be generated during incomplete combustion processes or formed in situ during atmospheric reactions.^6,7^ Such widespread presence means there is considerable interest in developing an improved understanding of their photophysics and photochemistry. This is particularly important given the possible health risks posed by both the parent compounds and their photochemical degradation products.^8,9^

In a recent publication,^10^ we reported a time-resolved photoelectron imaging (TRPEI) study of the nonadiabatic processes operating in the excited electronic states of nitrobenzene and three of its methyl-substituted derivatives following 267 nm excitation. The use of an intense 400 nm probe to induce significant 1 + 3′ ionization in these measurements offered an extended view of the overall photochemical reaction coordinate (with an effective probe energy of 9.3 eV). Despite this, however, we were unable to see any meaningful differences in the dynamics operating across all four species within the 200 ps observation window of our experiment. Three distinct temporal signatures were observed in our data, attributed to (i) an extremely rapid cascade of internal conversion steps leading from the initially prepared S_3_ (π_1_π_1_*) and S_4_ (π_2_π_1_*) states down through S_2_ (n_2_π_1_*) to the S_1_ (n_1_π_1_*) state (τ_1_ ≤ 30 fs); (ii) decay of the S_1_ (n_1_π_1_*) state via intersystem crossing (ISC) to the triplet manifold or competing IC directly to the S_0_ ground state (τ_2_ = 160–190 fs); and (iii) further ISC on a much more extended timescale connecting the lowest-lying state within the triplet manifold back to S_0_ (τ_3_ = 90–160 ps). Here, the state labeling notation follows that adopted by González and co-workers.^11^ This overall interpretation appears to be in broad agreement with mechanisms proposed in high-level quantum chemistry calculations undertaken for nitrobenzene^12−14^ and is consistent with findings reported in several other experimental studies using various different techniques and associated observables.^15−20^ The same calculations also indicated that the nonadiabatic dynamics within the excited state singlet and triplet manifolds are mediated by motions predominantly localized on the NO_2_ functional group. This appears to provide a rationale for the experimentally observed similarities between nitrobenzene and its methyl-substituted derivatives—despite considerable variations in starting molecular conformation prior to UV excitation. Our earlier findings also lead to the conclusion that elimination of both NO and NO_2_ photoproducts occurs on timescales in the nanosecond domain following a return of population to the vibrationally hot S_0_ ground state. Any differences in NO vs NO_2_ branching ratio upon site-selective methylation in nitroaromatic species^17,21,22^ are therefore likely to be a consequence of factors associated with dynamics occurring on this extended timeframe—including steric effects influencing isomerization prior to dissociation. We also highlight a very recent computational study by Giussani and Worth indicating that *ortho* methyl substituents and the enlargement of the π-system can enhance NO release through modification of the energy barrier connecting the T_1_ and S_0_ states, with the latter factor having a stronger effect.^23^

A wider review of background literature relevant to nitrobenzene photochemistry may be found in our earlier publication, and the reader is directed to that work for a more expanded perspective.^10^ A more recent article by Rodríguez-Córdoba et al. also provides an instructive perspective on the excited state dynamics and photochemistry operating in nitroaromatic species more generally.^24^ Here, we focus predominantly on a key discrepancy between our TRPEI findings and an ultrafast electron diffraction (UED) study reported by Zewail and co-workers.^25^ Following excitation at 267 nm, the UED data revealed a process operating in nitrobenzene with an exponential time constant of 8.8 ps. This was attributed to ISC and structural rearrangement of the T_1_ state leading to NO and phenyl radical products. Although the UED and TRPEI measurements were both conducted in the gas phase using the same pump wavelength, no dynamical signature operating on a timescale close to 8.8 ps was observed in the latter work. To try and resolve this discrepancy and gain further insight into the excited state photophysics of nitrobenzene, we have undertaken a new investigation employing ultrafast transient absorption spectroscopy (TAS). Once again using a pump wavelength of 267 nm, we now interrogate the dynamics operating in hexane and isopropanol solvents using a white light continuum probe (340–750 nm). The use of nonpolar hexane is expected to provide a good proxy to the gas-phase environment—particularly in relation to any femtosecond and few-picosecond processes (before the onset of significant solvent-induced vibrational relaxation). Results will therefore be instructive when compared directly to the UED and TRPEI findings. We note that similar transient absorption studies conducted on the structurally related 1-nitronaphthalene system in various solvents do show dynamics operating on timescales of a few picoseconds.^26,27^ This has been attributed to the decay of an intermediary member of the triplet manifold to the T_1_ state along with subsequent vibrational cooling. The ≥340 nm pump wavelengths used in these 1-nitronaphthalene studies were, however, significantly red-shifted from the 267 nm excitation that is employed here. Given that nitrobenzene is the prototypical nitroaromatic system, it is somewhat surprising to note that a study by Yip et al. from 1984 is the only TAS measurement so far reported.^20^ This work assigned a ≤5 ps lifetime for the S_1_ (n_1_π_1_*) state following 355 nm excitation in tetrahydrofuran, although the laser pulse duration (30–40 ps) and spectral observation window (415–650 nm) are limited in comparison to more modern measurements—particularly in regard to the temporal resolution. The relatively weak magnitude of the TAS signals reported from nitrobenzene has likely been a contributing factor in other, more recent time-resolved studies in the solvated phase making use of alternative transient grating or transient polarization techniques.^15,18,19^

## 2 Methods

### 2.1 Experimental Setup

**Figure 1:**
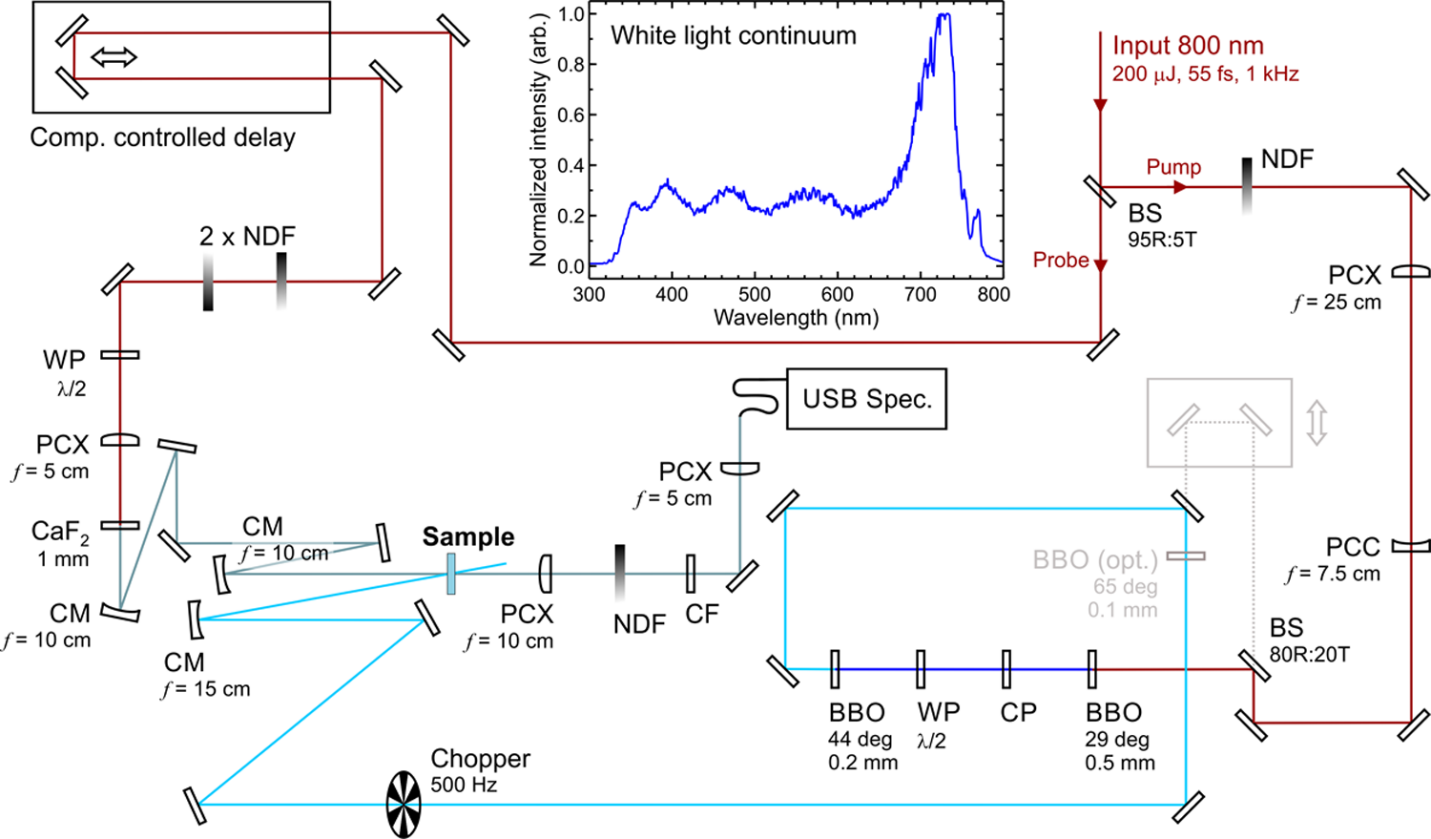
Schematic overview of the optical setup for transient absorption spectroscopy. Abbreviations: BBO (β-barium borate), BS (beam splitter, where *R* and *T* are reflectance and transmission percentages, respectively), CF (color filter), CM (concave mirror), CP (compensation plate), NDF (neutral density filter), PCX/C (plano-convex/concave lens), WP (waveplate). The gray-shaded beamline containing the BBO (opt.) component provides the possibility of generating a 200 nm pump as an alternative to 267 nm, although this was not used in the present study. The top inset shows a trace of the white light continuum generated inside the 1 mm CaF_2_ plate after passing through the color filter when the sample is removed.

Experiments were undertaken using a newly constructed transient absorption spectrometer that was developed in-house at Heriot-Watt University. A full description of the setup is therefore provided, and a schematic diagram may also be seen in Figure 1. A 200 mW portion of the fundamental 800 nm output from a 1 kHz Ti:sapphire oscillator/regenerative amplifier combination (Spectra-Physics Tsunami/Spitfire Pro) is initially split in a 95:5 ratio using a thin beam splitter (BS1-800-95-1012-45P, CVI Melles Griot). This provides the starting input for the pump and probe beamlines, respectively. After passing through a neutral density filter for power regulation (NDL-10S-4, Thorlabs), the pump beam (initially Ø = 1 cm) is reduced in diameter by around 70% using a pair of fused silica lenses forming a simple telescope (*f* = 25 cm and *f* = −7.5 cm). Frequency conversion of the pump beam to generate the third harmonic (267 nm) is initiated by passing the fundamental through a thin nonlinear β-barium borate (BBO) crystal to produce the 400 nm second harmonic. A calcite crystal then provides timing compensation between the 400 nm and residual 800 nm pulses. A dual λ/2 waveplate is subsequently employed to reorientate the polarization of the 800 nm beam prior to propagation through a second BBO crystal for the generation of the 267 nm pump. For the present experiments, this was typically limited to an energy of approximately 0.2 μJ pulse^–1^. Residual 400 and 800 nm pulses are removed from the 267 nm beam using a series of UV high reflectors.

In the probe beamline, 800 nm pulses are initially reflected off two right-angled mirrors mounted on a motorized linear translation stage (LTS300/M, Thorlabs) with 300 mm of total travel. Power control is achieved *via* a pair of variable neutral density filters (54–081, Edmund Optics) orientated inversely with respect to each other such that the propagating beam experiences a uniform intensity reduction. A λ/2 waveplate (WPH10M-808, Thorlabs) is incorporated to orientate the probe beam at the magic angle (54.7°) with respect to the pump, eliminating any time-dependent variation in absorption arising from rotational diffusion effects. The resultant pulses are subsequently focused by a fused silica lens (*f* = 5 cm) into a 1 mm CaF_2_ window (WCF-251, UQG Optics), where self-phase modulation produces a white light continuum (WLC) spanning the visible and near-UV spectral region. The CaF_2_ window is mounted on a piezo motor linear stage (CONEX-AG-LS25-27P, Newport) and kept in continuous motion to prevent optical damage (with a typical scan rate of 0.02 mm s^–1^). The WLC is then recollimated using a curved aluminum mirror (*f* = 10 cm). Pump and probe beams are subsequently focused using a pair of concave aluminum mirrors (*f* = 15 cm and *f* = 10 cm, respectively) and overlapped within a liquid flow cell (DLC-S25, Harrick Scientific Products). There is a minimum focused beam waist ratio of 3:1 between pump and probe (across the full spectral range), and a beam intersection angle of ∼6°. Given that the ionization potentials of nitrobenzene, hexane, and isopropanol all exceed 9.9 eV,^28,29^ the focused pump intensity (approx. 6 × 10^11^ W cm^–2^) is sufficiently low that the probability of generating any charged species is assumed to be negligible in our subsequent data analysis (as 2 × 267 nm photons only provide a total energy of 9.3 eV).

Circulation of liquid samples between a reservoir vessel and the flow cell is achieved using a small centrifugal impeller pump (M410K, TCS Micropumps) powered by an external variable DC voltage supply (IPS 2303, RS Pro). At the point of laser interaction inside the flow cell, the sample of interest passes between a pair of 2 mm thick CaF_2_ windows (WCF-252, UQG Optics) separated by a 100 μm thick PTFE spacer (MSP-100-M25, Harrick Scientific Products). Although not required in the present study, the flow cell is mounted on an additional piezo motor linear stage (CONEX-AG-LS25-27P, Newport) which may be used for sample translation during data collection to reduce the potential impact of bleaching and photoproduct deposition on the cell windows. Nitrobenzene (99.5%, Acros Organics) was prepared in hexane (≥95%, Fisher Scientific) and isopropanol (≥99.5%, Fisher Scientific) solvents at concentrations of 11.3 and 9.7 mM, respectively, and the flow rate through the cell set to 50 mL min^–1^. These concentrations were chosen based on preliminary data obtained using a commercial benchtop spectrophotometer (Shimadzu UV-2550) and ensured a linear absorption regime for the pump through the entire pathlength of the interaction region.

Following interaction with the sample, the WLC is recollimated using a fused silica lens (*f* = 10 cm) and passes through an optical color filter (740CFSP-19MM, Omega Optical) to cut out the intense 800 nm portion of the spectrum. The remaining probe beam is then spectrally analyzed by a 1 kHz compact spectrometer (AvaSpec-ULS2048CL-EVO-RS-UA, Avantes) via coupling into an optical fiber (M92L02, Thorlabs) using a further fused silica lens (*f* = 5 cm). The detector within the spectrometer consists of a 2048-pixel linear CMOS image sensor, providing a wavelength resolution of ∼0.7 nm across a 200–1100 nm spectral range. The combination of color filter and spectrometer yields a usable WLC between 340 and 750 nm, an example of which may be seen in Figure 1(inset).

### 2.2 Data Processing

**Figure 2:**
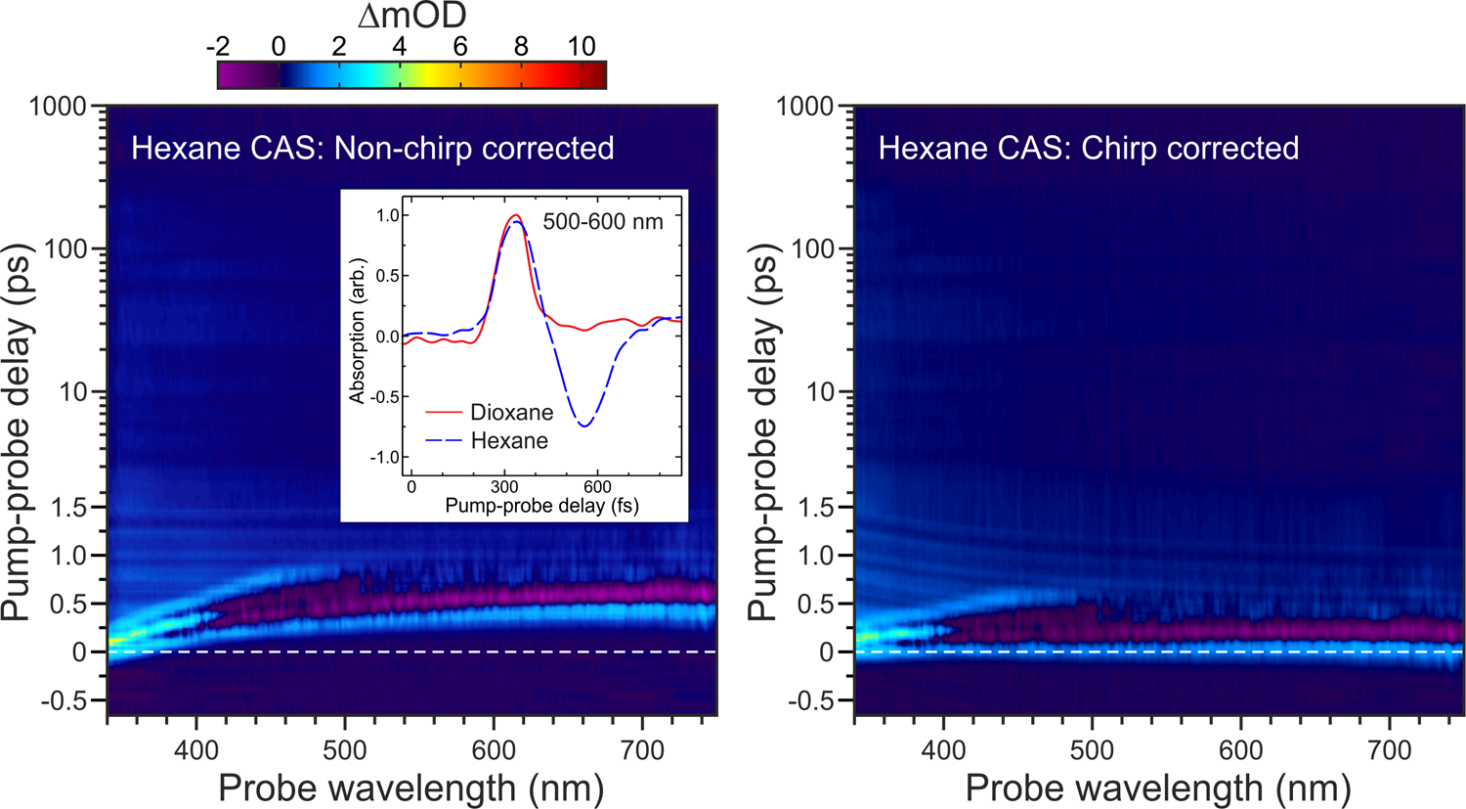
Hexane solvent coherent artifact signal (CAS) response under a 267 nm pump pulse. (Left) Raw transient data; (right) chirp-corrected data using the procedure described in the main text. For ease of direct comparison, the intensity color map here is identical to that used for the nitrobenzene TAS data presented in Figure 4. The inset in the left-hand-side panel shows an overlay of the hexane CAS integrated over the 500–600 nm range along with equivalent (intensity scaled) data obtained under identical experimental conditions for pure 1,4-dioxane. This confirms that the initial positive amplitude feature in the hexane CAS appearing at the earlier pump-probe delay times may be used to characterize the wavelength-dependent cross-correlation and chirp correction in subsequent TAS measurements. The very weak, wavelength-invariant modulations seen between 1.0 and 1.5 ps are an artifact arising due to a slight positioning instability in our delay stage.

The raw TAS data requires several processing steps to extract and quantify any molecular dynamical signatures. The accumulated pump-on and pump-off white light spectra must be compared to provide a measure of the change in absorbance of the system following UV irradiation. This is expressed as a differential change in optical density (ΔmOD) for each timestep interrogated
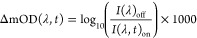
1Here, *I*(λ)_off_ is the time-invariant transmission spectrum of the ground state and *I*(λ,*t*)_on_ is the time-dependent transmission spectrum following photoexcitation (which contains contributions from both the ground and excited states). Spectra collected over a range of different pump-probe delay times Δ*t* display a nonlinear shift in the point of maximum temporal pulse overlap that increases toward longer wavelengths λ within the WLC. This is a consequence of chirping induced by transmission through optically dense media, and a correction for this effect must be applied prior to the extraction of any dynamical information. This is performed using the coherent artifact signal (CAS)^30^ obtained from a TAS measurement conducted on the pure solvents as shown for hexane in Figure 2(left). Additional measurements with an empty cell reveal this CAS response comes almost exclusively from the solvent rather than the CaF_2_ windows. For the case of both hexane and isopropanol under a 267 nm pump, the initial part of the transient response may be reasonably modeled by a Gaussian function *g*(Δ*t*,λ)
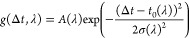
2Here, *A*(λ) is the amplitude of the Gaussian function at a given wavelength, *t*_0_(λ) is the wavelength-dependent central peak position in time, and σ(λ) is related to the peak full width at half-maximum (FWHM) via the relationship . This latter parameter also provides a wavelength-dependent cross-correlation for use in subsequent fitting analysis of our transient data. The validity of this overall strategy was confirmed by comparing the hexane transient signals with those obtained using 1,4-dioxane, a solvent that produces a purely Gaussian response right across the probe observation window^31^ (see Figure 2 (inset) for an example in the 500–600 nm region). More details on the nature of the overall shape of the CAS response may be found elsewhere.^32^ To ensure a smoothly evolving chirp correction is applied to the data, a set of independently fitted *t*_0_(λ) values are then supplied as inputs to the following expression
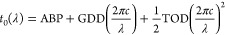
3where a global fit across all wavelengths yields an absolute phase term ABP, the group delay dispersion GDD, and third-order dispersion TOD. Before applying this chirp correction to our TAS data, however, some initial background processing is performed. First, a consistent offset reading from the spectrometer at each timestep is removed through subtraction of the average signal counts in wavelength regions outside the active observable range of the experiment. This subtraction is applied to both the solvent-plus-sample and solvent-alone spectra and is required as the overall integration time may differ across these two data sets. The solvent-alone transient may then subsequently be subtracted from the solvent-plus-sample data, producing TAS information that is free from background solvent signals. Although there are some known caveats with such a simple subtraction strategy (as absorption of the pump by the solute may modify the nonlinear solvent CAS response),^30,33^ in this instance the approach appears sufficiently robust to permit meaningful analysis of our data across all timescales. This is illustrated by the clean removal of CAS features seen in regions of our TAS data where there is no spectral overlap with the nitrobenzene probe absorption bands. Application of eq 3 then yields a chirp-corrected sample spectrum. This step is performed using linear interpolation to ensure that the same set of pump-probe delay times are provided at each wavelength within the overall data set (as this greatly simplifies subsequent fitting analysis). An example of this correction for hexane is shown in Figure 2(right).

## 3 Results

**Figure 3:**
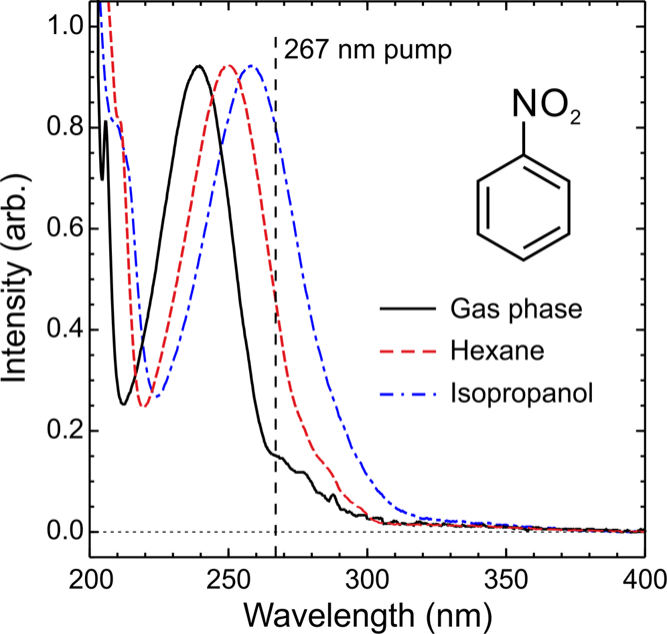
Room-temperature absorption spectra of nitrobenzene in the gas phase and in hexane and isopropanol solvents. Data were recorded using a commercial benchtop spectrophotometer (Shimadzu UV-2550). The vertical dashed line denotes the central 267 nm pump wavelength used in our TAS measurements. For ease of direct comparison, data have been scaled to the same intensity value at the maximum of the strong absorption band lying between 230 and 260 nm. Extending the wavelength range to the red of the 400 nm cutoff point (up to 800 nm) reveals no additional spectral features.

As already mentioned briefly in Section 2, preliminary room-temperature absorption spectra were obtained for nitrobenzene in both hexane and isopropanol over the 200–800 nm range in advance of commencing any TAS measurements. A section of these data is presented in Figure 3, along with the corresponding gas-phase spectrum that was reported in our previous TRPEI study. The strong absorption band with a maximum close to 240 nm in the gas phase arises from a transition to the S_4_ (π_2_π_1_*) state.^11−14^ This feature is red-shifted by approximately 10 nm in hexane and 20 nm in isopropanol. At the 267 nm pump wavelength used in our present measurements, however, we always excite at the long-wavelength side of this feature and some simultaneous direct optical preparation of the S_3_ (π_1_π_1_*) is also a possibility—as suggested in the nitrobenzene band simulations presented for the gas phase and in water by González and co-workers.^11^ Our preliminary absorption spectra also indicate that we should not expect any significant ground state probe (340–750 nm) absorption (and associated time-dependent bleaching effects) in our current TAS measurements.

**Figure 4:**
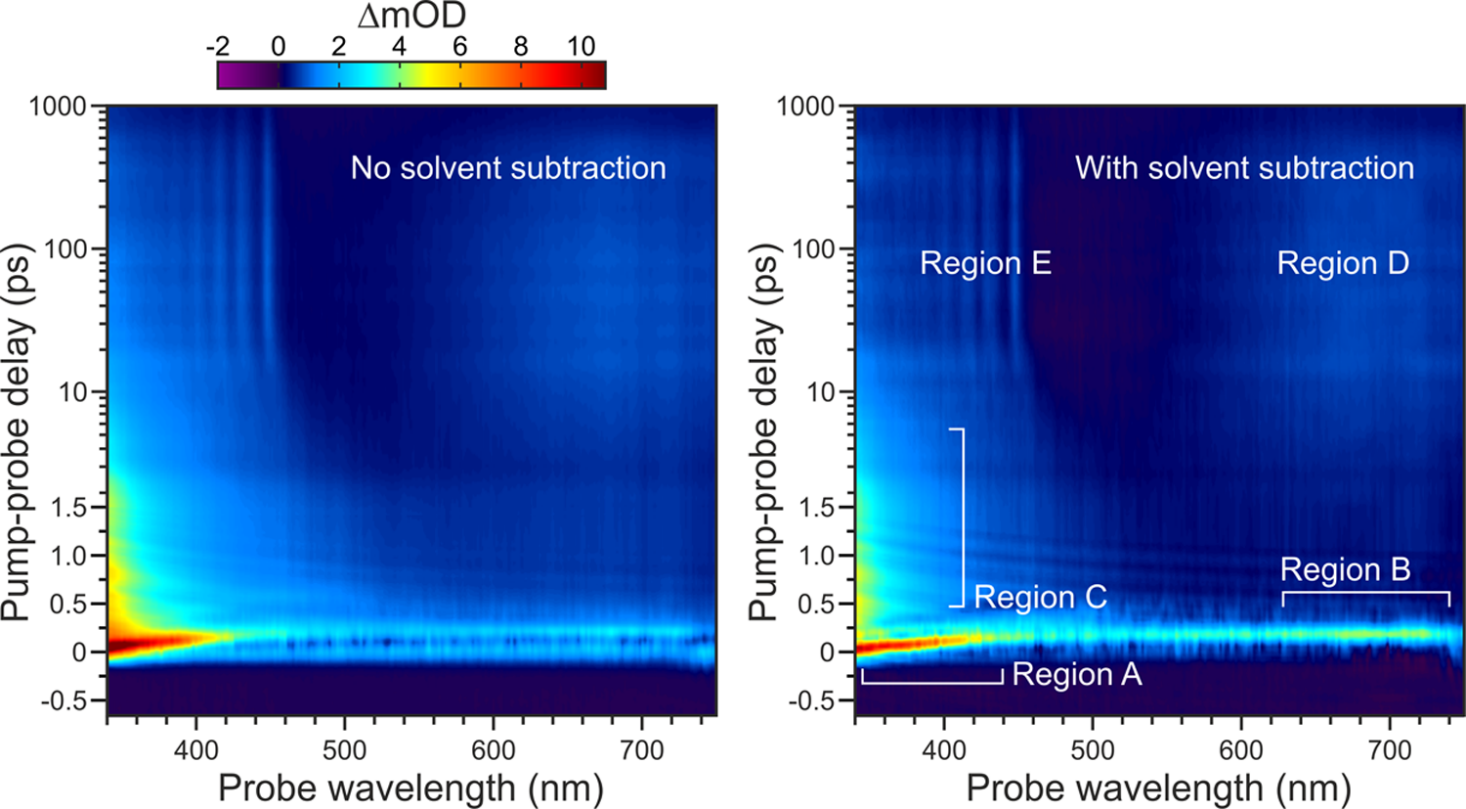
Chirp-corrected transient absorption spectrum of nitrobenzene in hexane under a 267 nm pump pulse. (Left) Without subtraction of the solvent background signal; (right) background subtraction applied (see Figure 2 for the associated solvent-only response data). Various regions of transitory behavior are highlighted, as discussed further in the main text. Note the mixed linear (−0.5 to 1.5 ps)–logarithmic (1.5 to 1000 ps) scaling of the pump-probe delay axis.

A chirp-corrected transient absorption spectrum obtained for nitrobenzene in hexane under 267 nm pump excitation is presented in Figure 4. Two versions of this plot are included to illustrate the importance of subtracting the solvent background. The data shows signals evolving on several different timescales within various distinct wavelength bands. To aid the subsequent discussion, key features are highlighted as Regions A-E. The first of these (Region A) is a relatively intense band that originates from zero pump-probe delay at the blue end of the spectrum (<400 nm). This band then appears to exhibit rapid (<200 fs) transitory evolution toward a second, weaker and extremely short-lived spectral band at much redder wavelengths (>660 nm, Region B). We stress here that this second band is a real feature in our data and not an artifact introduced by any imperfections in our probe chirp correction or in the subtraction of the solvent background signal. This is evident from the solvent-alone response information presented in Figures 2 and 4 (left) (where solvent background subtraction has not been applied). Returning to the blue end of the spectrum, an additional feature spans the approximate range 340–475 nm and exhibits a decay on the order of a few picoseconds (Region C). This decay appears correlated with a rising signal at the red end of the spectrum that leads to a broad and very long-lived band (Region D, >575 nm). Finally, the blue end of the spectrum also exhibits some very long-lived transitory behavior. This includes five narrow features forming a clearly discernible spectral comb that is characteristic of vibrationally resolved structure (Region E).

To numerically quantify the transitory dynamics, a multistep sequential exponential fitting model was applied to our data. This took the following general form
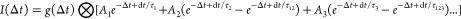
4Here, *g*(Δ*t*) is the predetermined Gaussian cross-correlation function, the *A*_*i*_ terms are amplitudes, d*t* captures any offset in the zero pump-probe delay position, and τ_12_ = τ_1_ τ_2_/(τ_1_ + τ_2_), τ_123_ = τ_12_ τ_3_/(τ_12_ + τ_3_), and so on. The FWHM of the *g*(Δ*t*) function varies between 130 and 170 fs at the red and blue extremes of the probe spectrum, respectively. Application of the model described by eq 4 to 25 nm wavelength-integrated slices of our transient data revealed broadly consistent time constants and associated fit amplitudes (of the same relative sign and approximate magnitude) across sizable blue (340–475 nm) and red (575–750 nm) probe wavelength bands. At the blue end of the spectrum, additional targeted regions were also investigated to confirm that elements of the vibrational comb feature seen in Figure 4 decayed on the same timescale as the underlying transient background signal. For both the 340–475 and 575–750 nm regions, a total of three sequential exponential functions were required to yield a satisfactory fit. For the purposes of further discussion, we label these functions using their associated time constants τ_1–3_, making a particular distinction between the shortest lifetime obtained in each region (i.e., τ_1A_ and τ_1B_ for the blue and red regions, respectively). In the wavelength region between these two limits (475–575 nm), no significant signal persists beyond 10 ps, and eq 4 could be limited to just two exponential terms. These exhibit similar transitory behavior to the first two terms used to model the blue end of the spectrum. As such, this intermediate wavelength region is not considered in further detail as it provides no additional information.

**Figure 5:**
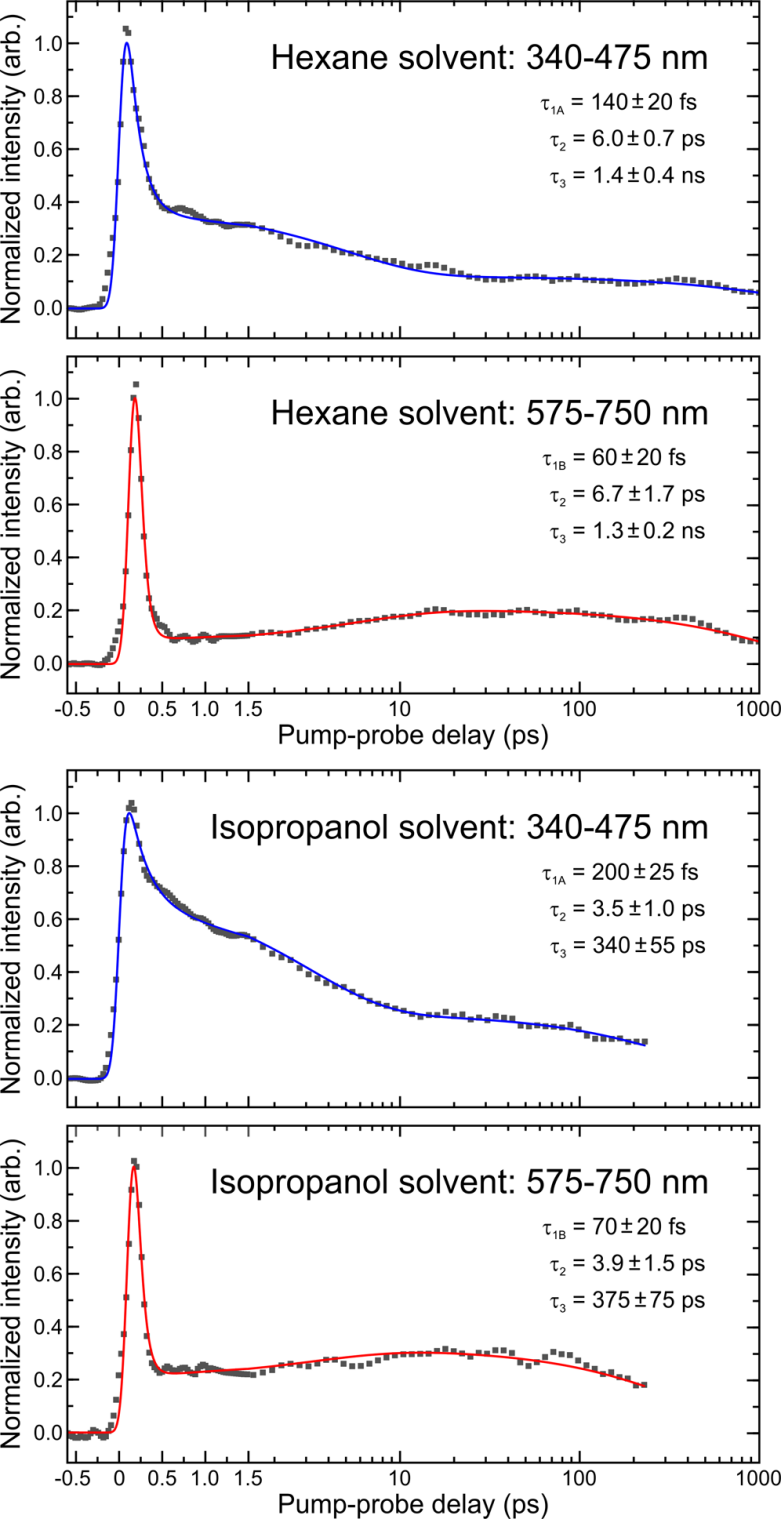
Data points show transient pump-probe signals obtained upon integrating the blue (340–475 nm) and red (575–750 nm) regions of the probe spectrum following 267 nm excitation of nitrobenzene in hexane and isopropanol solvents. Solid lines are the result of fitting to these experimental data using a three-step sequential model described by eq 4 given in the main text. Exponential time constants extracted from the fits are also included, along with 1σ uncertainties.

Figure 5 presents transient data for nitrobenzene in both hexane and isopropanol at the blue and red ends of the probe spectral observation window. Associated fits and numerical time constants are also included. Initially considering hexane, three decaying signals are clearly revealed in the blue (340–475 nm) region, operating on distinctly different timescales. The first of these (τ_1A_ = 140 ± 20 fs) describes an extremely rapid process, with the second (τ_2_ = 6.0 ± 0.7 ps) and third (τ_3_ = 1.4 ± 0.4 ns) components indicating the presence of much longer-lived dynamics. Of immediate note here is the lifetime associated with τ_2_, which is comparable to the 8.8 ps transient reported in the UED measurements of Zewail and co-workers—as highlighted in the Section 1. Turning now to the red (575–750 nm) component, an initial rapid decay is still observed (τ_1B_ = 60 ± 20 fs) but this is now offset by ∼100 fs from the Δ*t* = 0 pump-probe position. This reflects the slight temporal shift in the absorption band connecting Regions A and B seen in Figure 4. An exponential rise is then observed at longer pump-probe delay times (τ_2_ = 6.7 ± 1.7 ps). Within experimental uncertainty, this is well matched to the 6.0 ± 0.7 ps decay seen in the bluer spectral region. Finally, decay on a nanosecond timescale (τ_3_ = 1.3 ± 0.2 ns) then follows a very similar trend to that seen in the shorter-wavelength blue band. Upon switching the solvent from hexane to isopropanol, very similar overall behavior is observed in both spectral regions of interest, although the more extended dynamical timescales are somewhat shorter: In the picosecond domain, τ_2_ is reduced by an approximate factor of 2 (3.5 ± 1.0 and 3.9 ± 1.5 ps over the 340–475 and 575–750 nm regions, respectively), and this increases to more than a factor of 4 in the nanosecond regime (340 ± 55 and 375 ± 75 ps over the same selected wavelength bands). Previous studies investigating nonradiative relaxation of nitrobenzene in various solvents following 355 nm excitation reveal no obvious trend in lifetime *vs*. solvent polarity.^19^ In the related 2-nitrophenol molecule, however, a comparable shortening of the long-time (>100 ps) dynamics has been reported under isopropanol solvation compared to hexane at a 350 nm pump wavelength.^34^ In the case of the present data, a higher level of vibrational excitation in the initially prepared excited states of isopropanol vs hexane at 267 nm may be a contributing factor to the faster dynamical timescales—as suggested by the red-shifting of the absorption bands in the former solvent (see Figure 2).

**Figure 6:**
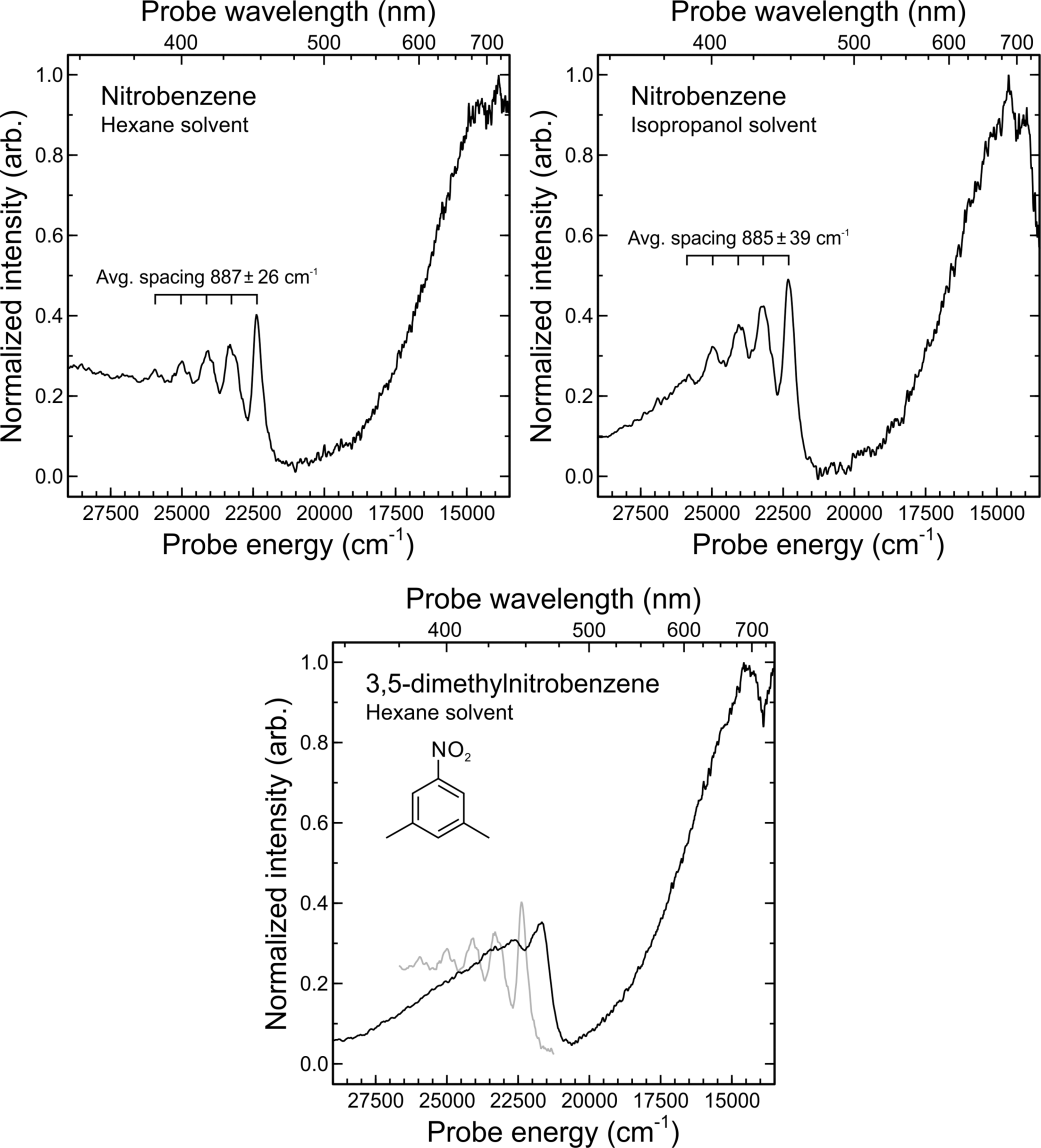
Top: Probe spectra integrated over the 20 ps to 1 ns time region (hexane solvent) (left) or 20–250 ps time region (isopropanol solvent) (right) following nitrobenzene excitation at 267 nm. An overlaid comb structure indicates the separation of 5 clearly discernable vibrational peaks. Bottom: Similar (exploratory) data obtained for the related 3,5-dimethylnitrobenzene molecule under hexane solvation within a 20–250 ps time window. For comparative purposes, a section of the nitrobenzene in hexane data is superimposed in gray. For additional details, see the main text.

To quantitatively investigate the vibrational comb structure seen in Region E of Figure 4, data were integrated over the 20 ps to 1 ns (hexane) or 20–250 ps (isopropanol) temporal regions and converted into an energy scale (with the application of an appropriate Jacobian intensity transform^35^). The spectra resulting from this process are shown in Figure 6(top). The average peak separation within the comb is 887 ± 26 and 885 ± 39 cm^–1^ for the hexane and isopropanol solvents, respectively. The absolute peak positions are also very similar in both cases, with isopropanol giving rise to a small red shift of approx. 50 cm^–1^ relative to hexane. Integrating the spectral data over smaller time intervals within the selected limits quoted above indicates that the relative peak amplitudes, positions, and widths within the comb structure do not change appreciably over the entire timeframe sampled in our measurements (as also suggested in Figure 4). This might indicate a very inefficient vibrational cooling mechanism for this specific vibrational mode, as expanded upon further in the next section. One obvious initial thought here is that the vibrational comb could be due to the ν_2_ bending vibration in an NO_2_ photoproduct (which could then, in principle, be observed to decay via germinate recombination). The peak spacing is, however, too large to be attributed to an extended progression in the NO_2_ X̃ state (ν_2_ ∼ 750 cm^–1^).^36^ The NO_2_*Ã* state, on the other hand, is known to exhibit a bending frequency much more closely matched to the peak spacing seen in our TAS data (ν_2_ ∼ 880 cm). Furthermore, the Ã←X̃ transition gives rise to a progression of several vibrational bands in the 370–460 nm region that align closely with the position of those seen in Figure 6.^37^ To further investigate this possible assignment, we conducted an exploratory TAS measurement on the related molecule 3,5-dimethylnitrobenzene under hexane solvation at extended pump-probe delay times (20–250 ps). The introduction of methyl substitutions at ring positions that are not directly adjacent to the nitro group is expected to exert only a negligible influence on the formation of NO_2_ photoproducts. It will, however, significantly alter many of the vibrational frequencies in the parent molecule relative to unsubstituted nitrobenzene. As seen in Figure 6(bottom), there is a loss of comb resolution and, more critically, a significant (∼15 nm) red-shifting of the one clearly identifiable peak feature. Such an observation would therefore appear to rule out NO_2_ photoproducts as the carrier of the vibrational spectrum.

## 4 Discussion

### 4.1 Femtosecond Dynamics (τ_1A_ and τ_1B_)

The observation of two extremely rapid time constants in our TAS data (as described by τ_1A_ and τ_1B_) is in general agreement with the behavior reported previously in our TRPEI measurements.^10^ We therefore attribute these to the same dynamical processes. Specifically, τ_1A_ corresponds to a cascaded decay of the S_3_ (π_1_π_1_*) and S_4_ (π_2_π_1_*) states initially populated under 267 nm excitation (Region A in Figure 4) down through S_2_ (n_2_π_1_*) to the S_1_ (n_1_π_1_*) state. The subsequent decay of the S_1_ (n_1_π_1_*) state via ISC into the triplet manifold and/or IC back to the S_0_ ground state (Region B in Figure 4) is then captured by τ_1B_. This is summarized in Table 1, along with the interpretation of the longer dynamical timescales discussed in subsequent subsections. The very short S_1_ (n_1_π_1_*) lifetime and ISC interpretation is broadly consistent with the extremely strong spin-orbit interaction predicted between the relevant states,^12−14^ as well as recent transient polarization spectroscopy data obtained from pure samples of liquid nitrobenzene following using a multiphoton 780 nm pump.^15^ We note here, however, that the wavelength *vs*. time curvature of the short-lived transient signal connecting Region A to Region B in our TAS data means that the actual numerical values attached to τ_1A_ and τ_1B_ in Figure 5 must be regarded as essentially qualitative. The key observation here, though, is the clear spectral resolution of two distinct mechanistic steps operating on an overall timescale of a few hundred femtoseconds.

**Table 1:** Summary of Experimental Time Constants and the Corresponding Mechanistic Interpretation for Nitrobenzene in Hexane (hex) and Isopropanol (ipa) Solvents Following 267 nm Excitation^a^^,^^b^

time constant	1/*e* lifetime	assignment
τ_1A_	<200 fs (hex/ipa)	S_4_/S_3_ → [S_2_] → S_1_
τ_1B_	<100 fs (hex/ipa)	S_1_ → T_2_ (or S_0_)
τ_2_	6.0–6.7 ps (hex)	T_2_ → T_1_
	3.5–3.9 ps (ipa)	
τ_3_	1.3–1.4 ns (hex)	T_1_ → S_0_
	340–375 ps (ipa)	

a See Figure 5 for associated uncertainties in specific quoted numerical values.

b Note that the wavelength vs time curvature of the short-lived transient signal presented in Figure 4 means that τ_1A_ and τ_1B_ only offer a qualitative guide.

### 4.2 Picosecond Dynamics (τ_2_)

In stark contrast to the dynamics operating in the femtosecond domain, the picosecond transient signal seen in our TAS data (τ_2_ = 3.5–6.7 ps) has no equivalent analogue in the earlier TRPEI measurements. As highlighted in Section 1, however, a UED study by Zewail and co-workers previously observed a process operating with a similar exponential time constant of 8.8 ps following excitation at the same 267 nm pump wavelength.^25^ This points to a potential limitation of the TRPEI approach, where the extent to which the probe projects into the ionization continuum impacts the overall view along the photochemical reaction coordinate.^38^ The properties of the cation state(s), in addition to the probe photon wavelength, may therefore exert a significant influence over the dynamical information that may be extracted from a given TRPEI measurement. An instructive starting picture here comes from Koopmans’ correlations, which point to a higher propensity for ionization processes connecting two states that exhibit similar molecular orbital configurations. As a simple example: a ππ* excitation based predominantly on a HOMO to LUMO + 1 transition would be expected to project strongly to the D_0_ (π^–1^) state of the corresponding cation. Alternatively, an nπ* excitation built predominantly on a HOMO – 1 to LUMO configuration should ionize preferentially into the D_1_ (n^–1^) continuum. These differences in ionization propensity will be strongly reflected in the energetic position of bands appearing in a time-resolved photoelectron spectrum when tracking the nonadiabatic population transfer between the ππ* and nπ* states. A more detailed discussion of Koopmans’ correlations in the analysis of TRPEI experiments may be found elsewhere.^39−41^

In the specific case of nitrobenzene, the first two electronic states of the cation, D_0_ and D_1_, are associated with ionization from delocalized π orbitals. In contrast, the D_2_ and D_3_ states correlate with the removal of an electron from orbitals exhibiting predominantly nonbonding character that are localized on the NO_2_ group.^28,42^ These four cation states have ionization thresholds of 9.94, 10.32, 11.01, and 11.23 eV, respectively.^28^ Within the framework of the (1 + 3′) ionization scheme in our earlier TRPEI measurements (for which the total photon energy was 13.94), all four of the lowest-lying singlet states—namely, S_4_ (π_2_π_1_*), S_3_ (π_1_π_1_*), S_2_ (n_2_π_1_*) and S_1_ (n_1_π_1_*)—can be detected efficiently. A similar argument should also apply for the triplet analogues of these singlet states. A key factor to note, however, is that quantum chemistry calculations also predict a low-lying state within the triplet manifold of nitrobenzene that is built on a dominant electronic configuration with no low-lying singlet analogue.^12,13^ This has been assigned as either T_1_ or T_2_, depending on the exact level of theory used, but the corresponding state within the singlet manifold sits at much higher energy (potentially S_5_ or above). A similar situation has also been reported in the case of 1-nitronaphthalene, where calculations conducted using time-dependent density functional theory indicate the T_2_ state is built on a HOMO-5 to LUMO transition.^43^ Once again, there is no low-lying singlet analogue of this state.

Given the observations outlined above, we therefore suggest that the T_2_ state in nitrobenzene preferentially ionizes to a cation state that sits energetically beyond the reach of the (1 + 3′) ionization scheme used in our earlier TRPEI study. This then rendered the measurement effectively blind to this specific step in the overall reaction pathway. Such a conclusion therefore leads us to now assign the (τ_2_ = 3.5–6.8 ps) lifetime seen in our TAS data to population moving through the T_2_ state, which is initially populated *via* ISC from S_1_ (n_1_π_1_*). This picture is consistent with more recent theoretical models developed for nitrobenzene by several different groups,^12−14^ as well as that proposed for the related 1-nitronaphthalene system.^26,27^ In our earlier work we highlighted that although decay back to S_0_ is expected to proceed from the lowest-lying T_1_ state, we were unable to directly comment on the potential dynamical role played by other members of the nitrobenzene triplet manifold. One scenario discussed was the possibility that the T_2_ state may decay to T_1_ on a timescale faster than the incoming transfer of population from the S_1_ (n_1_π_1_*) state via ISC. In this limit, any small (steady-state) levels of transient T_2_ population will not be efficiently detected.^44^ The observation of picosecond dynamics in our TAS measurements now appears to rule out this interpretation and instead leads us toward an explanation based on inefficient T_2_ ionization as a consequence of Koopmans’ correlations. A more detailed theoretical exploration of this possibility is beyond the scope of this present work but will form the basis of further investigations. Any future time-resolved experiments that monitor photoionization-based observables in nitrobenzene should therefore be conducted with probe photon energies that project much more deeply into the ionization continuum to provide a more complete map of the reaction coordinate. Examples of such measurements using XUV probes from free electron lasers^45,46^ and high-harmonic generation sources^47−50^ have previously been reported for several other molecular systems. This includes a study on the related 2-nitrophenol system, where picosecond dynamics were observed at relatively large electron binding energies following 260 nm excitation.^47^ Interestingly, a similar ionization-based measurement on the same system using 350 nm pumping in conjunction with a 400 nm multiphoton probe (as employed in our nitrobenzene TRPEI study) did not capture any picosecond component—although it was seen in accompanying transient absorption data recorded at the same excitation wavelength.^34^

### 4.3 Nanosecond Dynamics (τ_3_)

At both the red (575–750 nm) and blue (340–475 nm) extremes of the data presented in Figures 4–6, there are transient signals persisting all the way out to the temporal limit of our pump-probe observation window. These long-lived bands were also reported previously by Yip et al. in their aforementioned TAS study of nitrobenzene solvated in tetrahydrofuran.^20^ In this earlier work, the two features were observed to exhibit very similar kinetic behavior and were therefore both attributed to a single excited state transient absorbing in multiple wavelength regions. These authors also captured the first two (longest wavelength) peaks of the five-membered vibrational comb seen in Figures 4 and 6, although did not explicitly comment on their source. In our present measurements, the numerical time constants τ_2_ and τ_3_ associated with the long-time dynamics are very similar for both solvents considered across the respective blue and red absorption band regions (see Figure 5), also suggesting a common lower state in both cases. Furthermore, the transient decay on a picosecond timescale seen in the 340–475 nm region—which has already been assigned to the population leaving the T_2_ state—correlates with a rising signal between 575 and 750 nm. Once again guided by recent theoretical models,^12−14^ we therefore assign the long-lived τ_3_ signals seen in both the red and blue regions of our TAS data to decay of the nitrobenzene T_1_ state following its initial population *via* internal conversion from T_2_. To investigate this further, selected triplet excitations in hexane relative to the T_1_ minimum energy geometry previously published by Mewes et al.^13^ are presented in Table 2. This specific configuration is an appropriate starting point to consider given the extended lifetime of this state (>1 ns in hexane, > 300 ps in isopropanol). These data were obtained with the Gaussian16 software package^51^ using the equation of motion coupled cluster (EOM-CCSD) method in conjunction with the aug-cc-pVDZ basis set and the implicit SMD solvation model.^52^ The presence of two T_n_ ← T_1_ transitions with significant oscillator strength appear within our spectral observation window and are energetically well matched to the 575–750 and 340–475 nm absorption bands. A broadly similar prediction is also obtained from excited state data presented by Lin et al. using time-dependent density functional theory at the B3LYP/6-311(d,p) level with a T_1_ optimized geometry.^17^ The T_1_ state is believed to decay via ISC back to the S_0_ ground state before undergoing NO_2_ or NO elimination, where the relative branching yield of the two different photoproduct channels has been observed to vary considerably across the 320–193 nm excitation region.^17,21^ A recent theoretical investigation also suggests that the NO channel may potentially involve up to three different pathways.^53^ These fragmentation processes are, however, expected to occur on a timescale well beyond the limit of our current measurements.^17^ Finally here, we note that the T_1_ state lifetime (1.3–1.4 ns in hexane, 340–375 ps in isopropanol) is significantly longer than that attributed to the T_1_ state of nitrobenzene at the same 267 nm excitation wavelength in our earlier TRPEI study (90 ± 10 ps).^10^ Such a finding is not unexpected, however, given the potential for solvent-induced vibrational relaxation of various modes on relatively extended timescales that is not a factor in the gas phase.

**Table 2:** EOM-CCSD/aug-cc-pVDZ Vertical Triplet Excitation Energies and the Corresponding Oscillator Strengths Evaluated for Nitrobenzene Relative to the Optimized T_1_ Minimum Energy Geometry Reported by Mewes et al.^13^^a^

state	energy (eV/nm)	osc. str.
T_2_	0.29/4234	0.000
T_3_	1.52/818	0.012
T_4_	2.10/590	0.000
T_5_	2.22/558	0.000
T_6_	2.82/439	0.004
T_7_	3.85/322	0.000

a Hexane solvation effects were included using the implicit SMD model.

**Figure 7:**
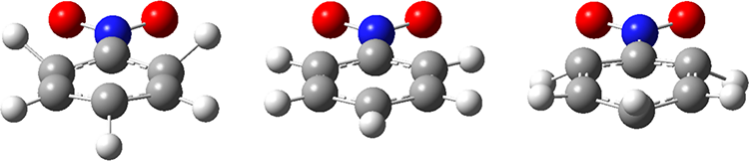
Illustration of the nitrobenzene vibrational mode potentially responsible for the 885–887 cm^–1^ comb spacing seen in Figure 6, as predicted by theoretical calculations described in the main text. The extreme positive (left) and negative (right) displacements are shown along with the equilibrium structure (center).

We now consider the vibrational comb structure seen within the 340–475 nm region of the TAS data, as highlighted in Figure 6. Given the assignment of τ_3_ to decay of the T_1_ state manifesting as two distinct absorption bands within our spectral observation window, it is perhaps initially surprising that the same comb is therefore absent in the 575–750 nm region. This could point to very different equilibrium geometries in the two different participating upper states, leading to an extended Franck-Condon progression in one absorption band but not the other. An alternative explanation, however, is simply that at the red end of the spectrum, the comb sits at absorption wavelengths >750 nm and is obscured by the optical color filter used to remove the intense 800 nm fundamental from which the WLC probe is derived. In future, experiments using an alternative driving frequency for supercontinuum generation may be used to further explore this suggestion. To learn more about the possible origin of the comb structure, we employed density functional theory (B2PLYP/Def2-TZVP) to determine the various vibrational frequencies present in the T_1_ state of nitrobenzene under isolated conditions, as well as in hexane and isopropanol. The B2PLYP method is well suited for problems requiring high accuracy in the evaluation of vibrational properties.^54^ Our analysis was once again conducted with the Gaussian16 software package at the optimized T_1_ minimum energy geometry, making use of the SMD solvation model. The key findings are presented in Table 3, which shows the computational result most closely matching that observed in our experimental data (885–887 cm^–1^) as well as the two nearest neighboring frequencies (one higher, one lower). All three modes correspond to large amplitude motion of the H atoms attached to the benzene ring with only minimal distortion/displacement of the NO_2_ group—as depicted for the most likely frequency carrier in Figure 7. There is no mode involving significant NO_2_ group displacement exhibiting a frequency within 100 cm^–1^ of the experimentally observed comb spacing. Given that the key motions driving the nonadiabatic dynamics throughout the excited state are known to be predominantly localized on the nitro moiety, this could potentially explain the relatively long lifetime of the T_1_ state, despite the apparent high levels of vibrational excitation within the 885–887 cm^–1^ mode. Furthermore, the apparent lack of vibrational quenching in this mode also indicates a highly inefficient resonant transfer of energy into the surrounding solvent bath. This is a surprising result as hexane, for example, is known to exhibit several vibrational modes with frequencies closely matched to the comb spacing.^55^ In light of this, we also reiterate the possibility that the observed vibrational progression may arise as a consequence of the upper state geometry being very different from that of T_1_. This could then give rise to an extended Franck-Condon progression in the absorption band that would not necessarily require any initial excitation in a specific T_1_ vibrational mode (and so would also not be subject to any solvent-induced quenching). The exact origin of the vibrational comb therefore remains an open question at the present time but may be investigated in future with more targeted measurements on systematically substituted nitrobenzene systems as well as more expanded theory.

**Table 3:** B2PLYP/Def2-TZVP Vibrational Frequencies Calculated at the T_1_ Minimum Energy Geometry for the Three Vibrational Modes of Nitrobenzene Closest in Energy to the 885–887 cm^–1^ Comb Spacing Seen in Figure 6^a^

vibrational frequency (cm^–1^|)		
gas phase	hexane	isopropanol
841.8	844.1	844.1
886.6	892.9	894.3
966.0	975.0	987.7

a No significant solvent shift is observed between hexane and isopropanol, consistent with our experimental observations. Also see Figure 7 for a graphical depiction of the 886.6–894.3 cm^–1^ mode.

## 5 Conclusions

A newly developed transient absorption setup has been used to investigate the nonadiabatic excess energy redistribution pathways exhibited by nitrobenzene in hexane and isopropanol solvents following 267 nm excitation. A white light continuum provides a probe spanning the 340–750 nm spectral region and this is sufficient to reveal dynamics operating with four individual exponential time constants. The first two of these are both of order 100–200 fs and are attributed to a rapid, cascaded decay of the initially populated S_4_ (π_2_π_1_*) and/or S_3_ (π_1_π_1_*) states (τ_1A_) down through S_2_ (n_2_π_1_*) to S_1_ (n_1_π_1_*)—which then undergoes ISC into the T_2_ state of the triplet manifold (τ_1B_) or internal conversion directly to the S_0_ ground state. The T_2_ state, which subsequently decays to T_1_ on a timescale of 3.5–6.7 ps (τ_2_), is known to be built on an electronic configuration that has no low-lying singlet analogue. This provides a rationale for the absence of dynamical signatures operating on picosecond timescales in a previous gas-phase TRPEI measurement at the same 267 nm excitation wavelength (using an effective probe energy of 9.3 eV). Our present findings also confirm the observation of picosecond dynamics in the UED study reported by Zewail and co-workers. Our interpretation of this signal (supported by recent theoretical developments reported by several groups) differs from this earlier work, however, as it does not involve isomerization of nitrobenzene to phenyl nitrate within the triplet manifold. Finally, the T_1_ state subsequently repopulates the S_0_ ground state on a nanosecond timescale under hexane solvation, and in approximately 350 ps when in isopropanol. Overall, our findings illustrate the value of drawing on a combination of complementary experimental measurements (with different associated observables) when attempting to understand the complete, and often highly complex nonadiabatic dynamics operating in polyatomic molecules.
